# Diagnostic study on major honeybee disease, pests and predators in North Western Ethiopia

**DOI:** 10.1002/vms3.1573

**Published:** 2024-08-01

**Authors:** Esubalew Shitaneh, Habtie Arega, Amssalu Bezabeh

**Affiliations:** ^1^ Ethiopian Institute of Agricultural Research Pawe Agricultural Research Center Pawe Ethiopia; ^2^ Oromia Institute of Agricultural Research Holeta Bee Research Center Holeta Oromia Ethiopia

**Keywords:** Amoeba, bee colony, disease, enemies, inspection, Nosema and Varroa

## Abstract

**Background:**

The study was conducted in Pawe district from Benishangul‐Gumuz and Jawi and Fagita Lekoma districts from the Amhara region to investigate major honeybee pests, predators and diseases.

**Methods:**

Using a purposive sampling technique, 183 households were interviewed, and 240 samples were collected for laboratory analysis of bee disease; data were analysed using descriptive statistics.

**Results:**

The share of hive types owned by sampled respondents was 88.6%; overall, 1.1% and 10.3% were traditional, transitional and modern beehives, respectively. About 92% of the sample respondents acquired their base colonies by catching swarm bees on the apex of trees. The majority of beekeepers executed external inspections of their colony, whereas only 50% carried out internal inspections. Based on the responses of beekeepers, around 48.9%, 56.3% and 23.1% of colonies absconded every year from Pawe, Jawi and Fagita Lekoma districts, respectively. Ants, wax moths, bee lice, beetles, spiders, birds, monkeys and honey badgers were the major honeybee pests and predators discovered in study areas in decreasing order. Concerning the incidence of Varroa mites, *Nosema apis* and amoeba disease, 27.5%, 60% and 71.6% of samples showed positive results in study locations, respectively.

**Conclusions:**

From this result, we observed that ants, wax moths, bee lice, beetles, spiders, birds, monkeys and honey badgers were the major honeybee pests and predators. The prevalence of amoeba disease was comparatively higher in highland areas and in the summer season. This finding suggests the need for the alertness of beekeepers in controlling bee disease and pests and strengthening bee colonies through seasonal colony management. There should be a strict quarantine, and check‐up undertaken when a new colony is purchased from one region to another is essential.

## Graphical abstract

Pests and predators are causing 55.8% of colony dwindling, 19.7% of absconding, 13.2% of death and 11.4% of honey loss in Pawe, Jawi and Fagita Lekoma districts.

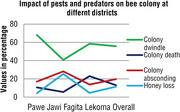

## 1 INTRODUCTION

Ethiopia is noticeable for its diversified agroecology and biodiversity, which makes the country ideal for the existence of a huge number of honeybee colonies (Nuru, 2007) and also makes the country the leading producer of honey and beeswax in Africa. Beekeeping remains one of the most profitable areas in the agricultural sector which has not yet been exploited to its full potential (Gebeyehu et al., 2010). It has multifaceted rewards and plays an important role in improving the productivity of food and cash crops and the conservation of natural resources through pollination. Moreover, beekeeping is a means of income for landless people living in harsh areas and also contributes to poverty reduction and foreign currency earnings through the marketing of honeybee products. However, the productivity of sector is falling due to the decline of the honeybee population; the influence of extensive use of agricultural chemicals and the arrival of pests, predators and diseases is of great concern to Ethiopia as well as many other countries (Abdulhay & Yonius, 2020; Central Statistical Agency, 2010; Kajobe et al., 2009).

Honeybee diseases, pests and predators are the obvious reasons for the death of honeybees, the consequent reduction in honeybee products and harm to domestic and international marketing of honeybee products (VanEngelsdorp & Meixner, 2010). Ethiopian agroecology is conducive for bees as well as honey pests, predators and microorganisms that are interrelating with the lives of honeybees. Diseases and the recent honeybee pests’ occurrence in Ethiopia, like chalk brood, small hive beetle and Varroa mites, have intensely manifested the importance of assessing the honeybee health and health risks (Begna, D., 2000 & 2006) in the country.

In Ethiopia, protozoa (Nosema and Amoeba), fungi (chalkbrood), parasitic mites (Varroa), small and large hive beetles, wax moths, ants, wasps, bee‐eater birds and honey badgers (Bezabeh, A. et al., 2012; Begna, D., 2015) were identified as major honeybee diseases and pests. Ants, wax moths, bee‐eater birds, praying mantis (Enziz in common name), honey badgers, bee lice, parasitic mites, small hive beetles, wasps, snakes and lizards are identified as the major causes of honeybee absconding (25.8%), dwindling and honey yield loss in east and west Gojjam zones of Amhara region (Ayele, B. et al., 2020). On the other hand, the incidences of bacterial and viral diseases, which are the most contagious ones, are not reported in the country (Abay et al., 2023). Thus, there is very limited knowledge of the current status, prevalence, distribution and economic importance of the existing diseases and pests on hand and the occurrence of non‐reported diseases. Therefore, it is a sense of control to study the status of current and the occurrence of non‐reported diseases, pests and predators of honeybees in the country in time to understand these troubles as early as possible and ensure sustainable beekeeping development. The objective of the current study was to identify the major honeybee diseases, pests and predators in the north‐western part of the country.

## 2 MATERIALS AND METHODS

### 2.1 Description of the study area

**FIGURE 1:**
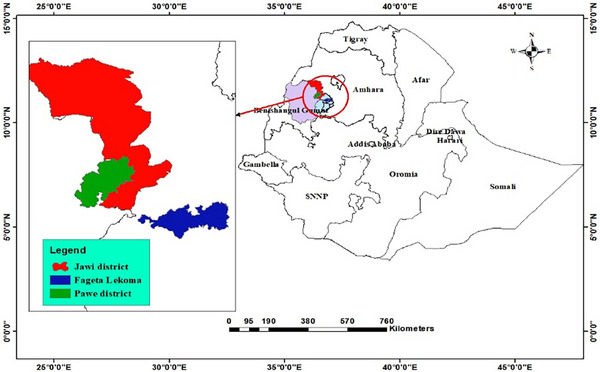
Map of the study area.

The diagnostic survey was conducted at three locations: Pawe district from the Metekel zone of Benishangul‐Gumuz and Jawi and Fagita Lekoma districts from Awi zone of Amhara region. Metekel zone is located between 80°45′ N–140 N latitude and 350°46′ E–400°25′ E longitude. Awi zone relies on 11°00′0.00″ N latitude and 36°39′59.99″ E longitude; the study area map is described in Figure 1. The study was conducted for three consecutive years, from 2020 to 2022 as shown in the (Table 1).

### 2.2 Sampling techniques and sample size determination

Before the actual survey was conducted, secondary data and baseline information were collected by consulting responsible extension officers at zonal and district levels. Based on the secondary data, semi‐structured questionnaires were prepared and pre‐tested. Based on their beekeeping potential and experiences, two zones, three representative districts and nine rural kebeles were selected. A total of nine kebeles, three kebeles at each district, were selected at a distance of 10 km between the adjacent sampling kebeles. The sample size of respondent beekeepers was determined based on sample size determination in the random sampling method for *N* (14,300) population of interest. Hence, a total of 183 beekeepers were selected for a diagnostic survey using random sampling methods, and it was disseminated uniformly to each of the selected apiaries. First, all beekeeper's populations were recorded in an excel sheet for each study kebele, and using a random sampling method, the study households were selected. Scientifically (Yemane, 1967), a sample size formula was used to calculate the sample size for this research:

n=N/1+N(e)2

Additionally, field observations were carried out on the presence of pests, and beekeepers were asked if any occurrences of diseases were observed, and their reports were recorded.

The sample size of inspected colonies was determined based on sample size determination in the random sampling method for an infinite population, using 50% of the expected prevalence of bee diseases and pests and a 95% confidence interval at 5% absolute precision. The number of bee colonies required and selected was 240 (Thrusfield, 2005) using the following formula:

$$n=\frac{196^{2}P_{\exp}\left(1-P_{\exp}\right)}{d^{2}}$$

where *n* is the required sample size; *P*_exp_ is the expected prevalence; *d* is the desired absolute precision.

### 2.3 Study design

A cross‐sectional study was conducted to assess the prevalence of common honeybee colony pests and diseases in the field through inspection and examination of samples at the laboratory for Varro mites, Nosema and amoeba disease using their respective protocols. Questionnaire survey was carried out during diagnostic study to determine honey production system and constraints caused by pests and predators. The prevalence for apiary and colony levels was calculated following the protocols of VanEngelsdorp et al. (2012):

$$\text{Prevalence}=\frac{\text{Number of positive cases}}{\text{Total number of sampled population}}\times 100$$

$$\text{Infestation level}=\frac{\text{Number of positive cases}}{\text{Total number of sample bees}}\times 100$$

A total of 240 honeybee colonies were selected, and samples of bees and brood combs were collected for observing the prevalence of honeybee disease and pests. The sample was collected during night‐time to reduce the disturbance of bees in the environment by wearing protective clothes. The samples of 10–20 adult worker honeybees and parts of brood comb were collected from the top of frames and hive entrances for testing for Nosema, Amoeba and Varroa mites, chalk brood disease, and American foulbrood (AFB) and European foulbrood (EFB) diseases. The collected samples were separated into transparent sample bottles preserved with 10% formalin and labelled immediately until laboratory analysis (Fries et al., 2013).

### 2.4 Laboratory examination of Varroa mite, chalk brood, American foulbrood and European foulbrood and Nosema and amoeba diseases

Varro destructor was subjected to laboratory examination, followed by the standard methods for Varroa detection (Dietemann et al., 2013). From each sample of honeybee colonies, 10 adult honeybees were brushed off from the brood comb and directly into a wide‐mouthed plastic container. The collected adult bees were killed using 70% ethyl alcohol and placed in 10 mL of a 1% detergent–water solution (10 mL detergent in 1000 mL water) and vigorously shaken for 1 min to dislodge mites. The mites were collected by filtering the solution through a ladle (8–12‐mesh) that held the bees back and let out the mites with the solutions. Then, wire gauze was used to hold the mites back and discharge the solutions. The wire gauze was turned down to white paper, on which the presence/absence of the mite was examined and counted. For brood examinations, samples 5 × 5 cm^2^ brood comb areas from drone and/or worker pupae broods were taken. About 100 pupae were randomly removed from their cells using forceps and checked for the presence of Varroa mites on the worker and/or drone pupae. The number of Varroa mites observed in both diagnoses (adult and brood) was recorded.

The chalk brood mummies were checked at the bottom board of hive entrance, in the comb cells and on the ground beneath the hive entrance. Mummies were moistened with distilled water, and the supernatant was placed on a microscope slid, covered with a cover slid and examined under a light microscope for spores and/or spore balls and cysts of *Ascosphaera apis*.

Field diagnostic procedures for AFB and EFB were used based on the OIE (World Organisation for Animal Health) (2008) procedure. During the early stages of decay until about 3 weeks after death, the dead larvae had a glue‐like consistency. To test for the AFB disease, larvae that must have discoloured, exhibit a melted appearance, ropiness, hard and dark scales that adhere strongly to the lower sides of the cell and protruding tongue were checked for their presence.

The laboratory examination of Nosema and Amoeba was undertaken using the following procedures: The sample bees were collected and preserved in 70% alcohol until laboratory analysis. The abdomen of honeybees from each sample was cut and ground in a mortar containing 5–10 mL distilled water. The mortar and pestle were thoroughly cleaned before being used again. A loop of suspension was placed on the microscopic slide using the sterilized loop and covered with a cover slide. The suspension was examined under a light microscope using 40 magnification powers for the presence of Nosema spores and Amoeba.

### 2.5 Data analysis

The collected data were coded, tabulated, analysed and interpreted using descriptive statistics of SPSS version 20. Descriptive statistics, such as means, mean deviation, frequency distribution, range and percentages, are presented in the form of tables and figures. Categorical data were collected, and analysis was done using descriptive statistics and the rank index formula as described by Musa et al. (2014). The chi‐square test was used to assess the association of the risk factors with the prevalence of the disease and pests. The differences were considered significant at (*p* < 0.05) with a 95% confidence level.

## 3 RESULTS

### 3.1 Demographic characteristics of households

The socio‐demographic characteristics of sample respondents are presented in Table 2. From the total of 183 sample households interviewed to generate qualitative and quantitative data on beekeeping, almost all were male. About 44.3% of household heads involved in beekeeping are between 20 and 45 years old, with an average experience of 4.69 years. Almost 99.5% of sample respondents had experience ranging from 1 to 9 years, but the rest only (0.5%) have above 10 years of beekeeping experience. Regarding educational status, around 53 respondents (29%) were illiterate, whereas 130 (71%) of households were found to be literate.

**TABLE 1:** Agro‐ecological information of the study districts.

Parameters	Pawe	Fagita Lekoma	Jawi
Latitude	11°18′40″–11°19′29″	10°57′–11°11′	10°57′17″–11°03′05″
Longitude	36°24′26″–36°25′27″	36°40′–37°05′	36°39′09″–36°48′25′
Altitude (m.a.s.l.)	1000–1200 m	1800–2950 m	500–1500 m
Mean Temp (°C)	24.56	24	17.7
Mean rainfall (mm)	1587	2371	1328

**TABLE 2:** Socio‐demographic characteristics of the respondents.

Number of sample respondent (*N* = 183)			
Demographic variables	Categories	Frequency	Percentage
Sex of respondent	Male	183	100
	Female	0	0
Age of respondent	20–45	81	44.3
	46–64	87	47.5
	Above 65	15	8.2
The marital status of the respondent	Single	7	3.8
	Married	173	94.5
	Divorced	2	1.1
	Widow	1	0.5
Educational status of the respondent	Illiterate	53	29
	Read and write	56	30
	Primary	25	13.7
	Junior	40	21.9
	Secondary	7	3.8
	Others diploma (10 + 3)	2	1.1
Beekeeping experience	1–5 years	99	54.1
	6–9 years	83	45.4
	Above 10	1	0.5

### 3.2 Honeybee colony management

The types of hives used in study areas were 1926 (88.6%) traditional hives, 23 (1.1%) transitional hives and 224 (10.3%) modern beehives, respectively, owned by sample respondents, as indicated in Table 3.

**TABLE 3:** Bee hives type and honeybee colonies at three districts in Metekel and Awi zones.

Districts	Bee colony in hive type			Total number of bee colony
	Traditional	Transitional	Modern	
Pawe	577 (93.8%)	1 (0.2%)	37 (6%)	615 (28.3%)
Jawi	1073 (88.5%)	14 (1.2%)	126 (10.4%)	1213 (55.8%)
Fagita	276 (80%)	8 (1.2%)	61 (10.4%)	345 (15.9%)
Total	1926 (88.6%)	23 (1.1%)	224 (10.3%)	2173

**FIGURE 2:**
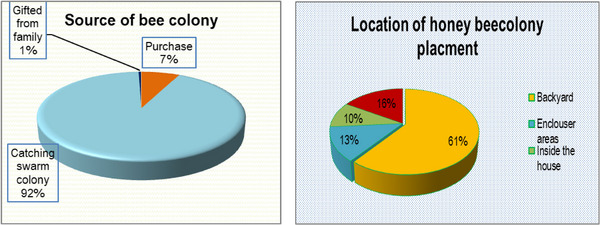
Source of bee colonies in proportion and colony placement.

About 92% of the sample respondents confirmed that they acquired their base colonies by catching swarms (hanging bait hives on the apex of trees), followed by 7% purchased from other beekeepers, and only 1% were required from parents by the gift presented in Figure 2.

About 61% of respondent beekeepers in the study area had placed their bee colonies in the backyard, whereas 16% were hung on the trees, 13% under the enclosure area, and only 10% prepared separate houses for their honeybee colonies, as the data shows in Figure 2.

**FIGURE 3:**
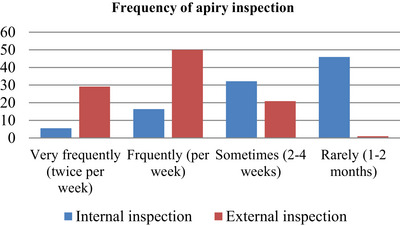
Frequency of external and internal colony inspection of sample respondent.

According to the data collected, 50% of respondent beekeepers inspected their colonies once a week, 29.1%, and 20.9% of the respondents took a look at external inspections twice per week and sometimes inspected their colonies, respectively. On the other hand, only 5.5% of respondents were inspecting internals twice per week, but 45.9% of respondents were inspecting their colony rarely (Figure 3).

### 3.3 Absconding, swarming and migration behaviour of the colony

Months of colony absconding, migration and swarming in different months of the year in the study area are shown in Table 4. Colony absconding and migration are two prominent behaviours of honeybee colonies; the absconding rate of a bee colony in Pawe, Jawi and Fagita Lekoma was 48.9%, 56.3% and 23.1%, respectively. On the other hand, the swarming tendency of bee colonies in study districts was 67.7% and 54.9% in Pawe and Jawi districts during October, and 67.7% in Fagita Lekoma during November.

**TABLE 4:** Percentages of colony absconding, migration and swarming in different months.

		Months of colony absconding							
Districts	*N*	September	October	November	December	January	February	March	April
Pawe	47	–	6.4	10.6	6.4	48.9	12.8	6.4	8.5
Jawi	71	7	5.6	2.8	14.1	56.3	7	7	–
Fagita Lekoma	59	1.5	3	10.5	7.7	23.1	23.1	7.7	13.9
Total	183	8.5	16	23.9	–	–	–	–	–
Months of colony swarming									
Pawe	47	19.1	61.7	12.8	6.4	–	–		
Jawi	71	1.4	54.9	36.6	4.2	1.4	1.4		
Fagita Lekoma	65	–	23.1	67.7	9.2				
Total	183	–	–	–	–	–	–		
Months of colony migration									
Pawe	47	–	8.5	–	6.4	53.2	25.5	4.3	2.1
Jawi	48	1.4	12.7	7	9.9	28	4.2	48.2	
Fagita Lekoma	30			4.6	3.1	21.5	6.2	6.2	4.6
Total	183								

### 3.4 Major honeybee pests and predators

Different honeybee adversaries cause bee colonies’ ailment directly and indirectly; the reaction of sample beekeepers from study districts on the incidence of major honeybee pests and predators is shown in Table 5. The major honeybee pets and predators mentioned were ants, wax mouth, bee lice, beetles, spiders, birds, monkeys and honey badgers, where the major enemies were worsening for bee colonies in the study areas.

**TABLE 5:** Major honeybee pests and predators in the sampled district.

Honeybee pests and predators	The relative degree of importance according to the respondent									Overall rank
	1st	2nd	3rd	4th	5th	6th	7th	8th	Index	
Ants (*Dorylus fulvus*)	48	59	34	18	12	9	0	0	0.173	1
Wax moth (*Achroia grisella*)	51	63	27	11	9	3	0	0	0.165	2
Honey badgers (*Mellivora capensis*)	45	29	24	19	29	6	0	0	0.140	3
Bee‐eater birds (*Meropidae*)	30	34	34	21	3	6	0	7	0.122	5
Monkeys	40	41	22	30	11	0	6	0	0.130	4
Bee lice (*Braula coeca*)	31	41	20	10	8	4	3	0	0.112	6
Small hive beetles (*Aethina tumida*)	11	18	15	20	20	21	11	10	0.086	7
Spiders (*Arachnids*)	23	15	12	19	15	6	5	2	0.081	8

*Note*: Index = sum of (8*ranked 1st + 7* ranked 2nd + 6* ranked 3rd + 5* ranked 4th + 4* ranked 3rd + 3* ranked 2nd + 2*ranked 1st ranked) for individual pests and predators divided by the sum of (8*ranked 1st + 7* ranked 2nd + 6* ranked 3rd + 5* ranked 4th + 4* ranked 5th + 3* ranked 3th + 2*ranked 2nd + 1* ranked 1st + 1*) for overall pests and predator.

**FIGURE 4:**
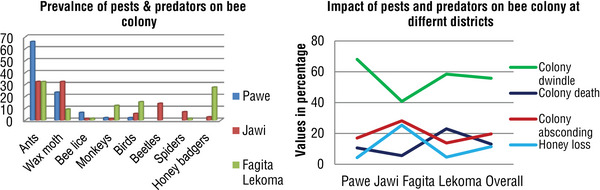
The prevalence of pests and predators in sampled districts and different season.

In this study, all sampling localities, pests and predators have been reported as a severe problem of beekeeping that affects about 55.8%, 19.7%, 13% and 11.4% of colonies; dwindling, absconding, death and loss of honey are shown in Figure 4.

### 3.5 Laboratory diagnosis of honeybee pests and diseases

#### 3.5.1 Prevalence of Varroa mites (*Varroa destructor*)

According to our survey results, the Varroa mite has been observed in 29.24%, 26.78% and 25.64% of the samples that were positive in Pawe, Jawi and Fagita Lekoma districts, respectively, and overall, 27.5% of samples had positive results, as shown in Table 6.

**TABLE 6:** Prevalence of Varro mite and its risk factors on honeybees in sampled districts.

Observed risk factors	Categories	Number of hives examined	Varro prevalence		Chi‐square	Likelihood ratio	*p*‐Value
			Positive	%			
Location	Pawe	106	31	29.24	0.311	0.311	0.856
	Jawi	56	15	26.78			
	Fagita Lekoma	78	20	25.64			
Mean		240	66	27.5			
Season	Dry season	120	26	21.67	4.096	4.12	0.0
	Summer	120	40	33.33			
Mean		240	66	27.5			

#### 3.5.2 Prevalence of Nosema disease (*Nosema apis*)

The occurrence of the Nosema incident in the study location is shown in Table 7. Nosema is caused by *Nosema apis* and *Nosema ceranae*. It is a microsporidian fungal disease that infects the intestinal tract of adult bees. The laboratory diagnostic result from 240 honeybee colonies confirmed that the incidence of *N. apis* was 60% (144) samples showing positive results at Awi and Metekel zones. Based on laboratory results, there was no significant difference in the incidence of Nosema among seasons, but 64.16% of cases were observed in summer, and 55.83% were in the dry season.

**TABLE 7:** Prevalence of Nosema and its risk factors on honeybees in sampled districts.

Observed risk factors	Categories	Number of hives examined	Varro prevalence		Chi‐square	Likelihood ratio	*p*‐Value
			Positive	%			
Location	Pawe	106	67	63.2	3.046	3.006	0.218
	Jawi	56	28	50			
	Fagita Lekoma	78	49	60.82			
Season	Dry season	120	67	55.83	1.736	1.739	0.236
	Summer	120	77	64.16			
Total		240	144	60			

#### 3.5.3 Prevalence of amoeba disease (*Malpighamoeba mellificae*)

The manifestation of Amoeba (*Malpighamoeba mellificae*) in the study districts is presented in Table 6. A total of 240 honeybee colonies were assessed for the existence of amoeba disease; from this, 71.6% (134) samples had positive results.

## 4 DISCUSSIONS

### 4.1 Household's characteristics

Of the total sample households interviewed, all were male; they headed beekeeping activities. The result agrees with Mujuni et al. (2012). This might be because females in beekeeping activities are too much, but these are reported as the work of men (head of the household). The average age of beekeepers in the current result was 27–45 years old. The majority of beekeepers have 1–9 years of beekeeping experience, are productive and are actively involved in beekeeping activities. The present result was complementary to the result of Jatema, D. & Abebe, B. (2015) around (61.34%) of respondents had between 1 and 10 years of experience in beekeeping. Regarding educational status, the majority of respondents learned, which shows they are actually to receive honeybee keeping technology. The present result was lower than those reported by Kinati, C. et al. (2013). Overall, 34.4% from the Lasta district and Tezera (2013) 33.57% of respondents in the Gomma district were illiterate.

### 4.2 Honeybee colony management

According to the survey results, about 88.6% of beehives were traditional, 11% were transitional and 10.3% were modern beehives owned by the sampled respondents. When compared to the three districts, Pawe district has a very low adoption of modern hives; this might be because the majority of beekeepers in the Metekel area practice a forest beekeeping system. The recent result was in line with the 9.3% of modern hives reported by Segni (2017) from the Ejere district.

About 92% of the sample respondents confirmed that they acquired their base colonies by catching swarms (hanging bait hives on the apex of trees); this disagrees with the previous report of Adane et al. (2020), which reported that 35.0%, 31.7% and 33.3% of respondents started beekeeping through purchasing, keeping through catching swarms and getting hives from their parents, respectively. This might be because the Metekel and Awi zones are rich in flora diversity as well as the large number of bee colonies easily available.

About 61% of respondent beekeepers in the study area had placed their bee colonies in their backyards. The current result was that less than (78.6%) of beekeepers in western Amhara regions placed their honeybee colonies in their backyards (Abay et al., 2023). This might be because the majority of the north‐western part of honeybees is aggressive, and their high migratory behaviour inclines bee colonies difficult to familiarize in the backyard.

According to the data collected, 50% of respondent beekeepers inspected their colony once a week, and only 5.5% of respondents inspected internal inspections twice per week. Our current result is higher than the previous result of only 83.3% of the respondents at Bale zone inspecting their apiary sites (Gelgelu, T. & Tesfaye, B., 2023).

### 4.3 Absconding, swarming and migration behaviour of the colony

Colony absconding and migration are two prominent behaviours of honeybee colonies; the absconding rate of a bee colony in Pawe, Jawi and Fagita Lekoma was 48.9%, 56.3% and 23.1%, respectively. The disease, seasonality of bee flora and environmental fluctuations were the major factors that initiated bee colony absconding. This is in good agreement with the previous report of Shitaneh et al. (2022), who discovered that the main reasons for absconding were pests and predators, environmental flux and disturbance during bee colony manipulation.

The swarming tendency of bee colonies in study districts was 67.7% and 54.9% in Pawe and Jawi districts during October, and 67.7% was in Fagita Lekoma during November. The recent study was in agreement with the previous finding of Shitaneh et al. (2022), indicated that *Apis mellifera scutellata* honeybees had a high reproductive swarming tendency; on average, 3.39 ± 0.6 queen cells per colony were observed.

### 4.4 Major honeybee pests and predators

Different honeybee adversaries cause bee colonies’ ailments directly and indirectly. The major honeybee pets and predators mentioned in the study area were ants, wax mouth, bee lice, beetles, spiders, birds, monkeys and honey badgers; the major enemies were worsening for bee colonies in the study areas. According to the response of beekeeper's major cause on bee colonies and hive products, the ant is the foremost predator that attacks honeybee colonies in three districts, and the wax moth is the second one from Pawe and Jawi districts, but the honey badger was ranked as the second‐affecting colonies from Fagita Lekoma district. The sample respondent survey result indicates that the intensity of pest and predator loss in different districts is significantly different. But bee lice have the slightest impact on the honeybees in the Jawi and Fagita Lekoma districts; in other words, beetles, spiders and honey badgers do not affect honeybees in the Pawe district. The result is also in line with Ayele et al. (2020), in which similar kinds of pests and predators were observed and the frequency of each type of pest and predator was similar.

In this study, in all sampling localities of the survey districts, pests and predators have been reported as a severe problem of beekeeping that affects about 55.8%, 19.7%, 13% and 11.4% of colonies, dwindling, absconding, death and loss of honey. The damage to pests and predators on honeybees varied based on the season and agroecology of the area; according to the interviewed beekeepers, the most serious damage happened during summer, followed by autumn and winter. Our result was divergent from the report of Jatema, D. & Abebe, B. (2015); at surrounding Finfine in Walmara district, destruction of pests and predators was observed during autumn, spring, summer and winter seasons in decreasing order. This might be the difference in agroecology and the type of presence of different pests and predators in different areas.

### 4.5 Laboratory diagnosis of honeybee pests and diseases

#### 4.5.1 Prevalence of Varroa mites (*Varroa destructor*)

According to our survey results, the Varroa mite has been observed in 29.24%, 26.78% and 25.64% of the samples that were positive in Pawe, Jawi and Fagita Lekoma districts, respectively, and overall, 27.5% of the samples had positive results. Regarding agroecology, the highest Varroa manifestation occurred during the summer season. The population of Varroa mites during the dry season and low brooding season was low, and the growth of mites’ brood population depends on honeybee brood production (Begna et al., 2016). The present finding was higher than 18.06% of positive Varroa mites reported from Bale zone (Gelgelu, T. & Tesfaye, B., 2023), and conversely lower than 82%, 85.9% and 36.5% of Varroa prevalence reported from Tigray, eastern parts of the Amhara region and Southwest Ethiopia (Begna, 2014; Tsegaye, A. et al., 2015; Tulu et al., 2023), respectively. The substantial variances between different reports were mainly due to the deviations in climate conditions, beekeeping systems, types of hives used, placement sites and the race of bees; this was supported by Pirk et al. (2016), Shegaw & Bezabeh (2023) and Shitaneh et al. (2022).

#### 4.5.2 Prevalence of Nosema disease (*Nosema apis*)

According to the laboratory investigation, Nosema and amoeba diseases have made possible identification and verification in both broods and combs, but other diseases have not been observed. Even in suspected cases of abnormal brood, the Nigrosine test was negative for both brood diseases. The occurrence of the Nosema incident occurred in the study location. Nosema is caused by *N. apis* and *N. ceranae*. It is a microsporidian fungal disease that infects the intestinal tract of adult bees. About 60% of the sample showed positive results at Awi and Metekel zones. Based on laboratory results, the incidence of Nosema showed a significant difference among seasons; overall, 64.16% of cases were observed in summer, and 55.83% were in the dry season. The present result was in line with the earlier report of Aster et al. (2007), in which 60% of Nosema positive results were observed in Benishangul‐Gumuz region. However, more than 53.3%, 58% and 47% were reported in Addis Ababa, Oromia and Amhara regions (Nega et al., 2019; Yohanis et al., 2010; Begna, D. & Kebede, Y., 2005), respectively. But lower than the 88%, 95%, and 60% of incidence rates reported in Oromia, Amhara and Benishangul‐Gumuz regions (Aster et al., 2007), respectively. The main reason for the variability of the report might be due to the difference in geographical location; the area that is more humid is conducive to the multiplication of fungi.

#### 4.5.3 Prevalence of amoeba disease (*Malpighamoeba mellificae*)

Amoeba is a disease of honeybees caused by a single‐celled parasite called *M. mellificae*, which affects the Malpighian tubules of honeybees and shortens their life cycle. The diseases were reported in Ethiopia, together with Nosema, with low infestation levels (Begna, D. & Kebede, 2005). Overall, 71.6% of samples showed amoeba disease‐positive results. The current research result was lower than the value (82.3%) of the sample, which was a positive result in Ejere district (Segni, 2017). The present result indicated that the incidence of amoeba disease was comparatively higher in the Fagita Lekoma district (58.9%) than (in Jawi) (51.88%). This shows that amoeba disease is more prevalent in all aspects than the other honeybee diseases, even if Nosema and amoeba diseases are more prevalent in the rainy season (Ayele et al., 2020). The differences in prevalence between seasons were statistically non‐significant (*χ* ^2^ = 3.31; *p *= 0.091), as shown in Table 8, but numerically, the summer season is more affected than the dry season. This might be related to the summer season being conducive to the reproduction of the disease. The current result is in line with the findings of Bezabeh and Begna (2012), who reported that the highest disease intensity was recorded in April and August high humidity and the lowest intensity in January high temperature.

**TABLE 8:** Prevalence of Amoeba and its risk factors on honeybees in sampled districts.

Observed risk factors	Categories	Number of hives examined	Amoeba prevalence		Chi‐square	Likelihood ratio	*p*‐Value
			Positive	%			
Location	Pawe	106	56	51.88	0.739	0.739	0.691
	Jawi	56	32	57.14			
	Fagita Lekoma	78	46	58.97			
Season	Dry season	120	60	49.16	3.31	3.32	0.091
	Summer	120	74	94.1			
Total		240	134	71.6			

## 5 CONCLUSION AND RECOMMENDATIONS

In conclusion, about 92% of the sample respondents acquired their base bee colonies by catching swarms by hanging bait hives on the branches of trees. The majority of beekeepers inspect their colonies’ externals every 1 and 2 weeks, but only 50% perform internal check‐ups of their colonies. Ants, wax mouths, bee lice, beetles, spiders, birds, monkeys, and honey badgers were the major honeybees’ pests and predators well familiar in the study areas. Ant is the foremost predator that attacks honeybee colonies in three districts, followed by wax moth. Pests and predators have been reported as a severe problem that causes around 55.8% of colonies dwindling, 19.7% absconding, 13% to die, and 11.4% of honey loss in study districts. On average, around 27.5% of samples have been observed to be Varroa mite‐positive and 60% of *N. apis*–positive results in study districts. On the other hand, about 64.16% of *N. apis* cases were observed in summer, and the rest were 35.83% in the dry season. From 240 honeybee colonies assessed for the existence of amoeba disease around 71.6% of samples had positive results. The result indicated that the incidence of amoeba disease was comparatively high in highland areas and mostly ascended in the summer season. The result suggested that the alertness of beekeepers in controlling bee disease and pests through strengthen bee colonies, improving seasonal colony management and conducting regular inspections. A strict quarantine and check‐up undertaken when a new colony is purchased from one region to another is essential.

## AUTHOR CONTRIBUTIONS

**Esubalew Shitaneh**: Conceptualization; investigation; funding acquisition; writing—original draft; methodology; software; data curation; supervision; resources; formal analysis; visualization; validation; writing—review and editing; project administration. **Habtie Arega**: Funding acquisition; methodology; writing—review and editing; resources. **Amssalu Bezabeh**: Project administration; writing—review and editing; methodology; conceptualization; funding acquisition.

## CONFLICT OF INTEREST STATEMENT

The authors declare no conflicts of interest.

## CONSENT FOR PUBLICATION

All authors decided on the publication of this paper and held the corresponding author accountable for correspondence during manuscript publishing.

## ETHICS STATEMENT

Not applicable.

### PEER REVIEW

The peer review history for this article is available at https://publons.com/publon/10.1002/vms3.1573.

## Data Availability

The data that support the findings of this study are available from the corresponding author upon reasonable request.
